# Role of Cell‐Cycle Proliferation Test, Triple Hit Phenotype, and TMPRSS2‐ERG Expression to Evaluate the Risk of Progression in Prostate Cancer Patients Under Active Surveillance

**DOI:** 10.1002/pros.24921

**Published:** 2025-05-29

**Authors:** Marco Oderda, Giulia Orlando, Giorgio Calleris, Giulia Capella, Luisa Delsedime, Eleonora Duregon, Paola Francia di Celle, Donatella Pacchioni, Ian Marc Bonapace, Zaibunnisa Zaibunnisa, Daniele D'Agate, Gabriele Montefusco, Claudia Filippini, Mauro Papotti, Paolo Gontero

**Affiliations:** ^1^ Division of Urology, Department of Surgical Sciences, Città della Salute e della Scienza di Torino, Molinette Hospital University of Turin Torino Italy; ^2^ Pathology Unit, Department of Oncology University of Turin, Città Della Salute e Della Scienza di Torino Turin Italy; ^3^ Pathology Unit, Department of Medical Sciences University of Turin, Città Della Salute e Della Scienza di Torino Turin Italy; ^4^ Pathology Unit Città della Salute e della Scienza di Torino Turin Italy; ^5^ Department of Biotechnology and Life Sciences University of Insubria Busto Arsizio (Va) Italy; ^6^ Department of Surgical Sciences University of Turin Turin Italy

**Keywords:** active surveillance, cell‐cycle progression, ERG, progression, Prolaris, prostate cancer, PTEN, TMPRSS:ERG, triple hit

## Abstract

**Background:**

An accurate estimation of progression risk in patients with prostate cancer (PCa) amenable to active surveillance (AS) is still an unmet need. Among available biomarkers, we considered Prolaris cell‐cycle progression (CCP) test, “triple hit” phenotype (ERG overexpression, PTEN and prostein expression loss) and elevated expression levels of TMPRSS2‐ERG gene fusions.

**Methods:**

We performed a case‐control study, enrolling patients that entered the AS programme at our tertiary referral Institution. Men subsequently undergoing radical prostatectomy for progression were considered as “cases”, while men still on AS at the end of the follow‐up period were labeled as “controls”. CCP test, triple hit and TMPRSS2‐ERG expression analyses were performed on tumoral tissue retrieved from biopsies at enrollment. Their ability to distinguish “cases” and “controls” was evaluated. According to power analysis, the study required 40 patients.

**Results:**

Patients had comparable baseline characteristics. CCP test suggested to continue AS in 75% of controls and to undergo an active treatment in 75% of cases. CCP molecular score (HR 8.5, *p* = 0.02) was significantly associated with progression in multivariable logistic regression. No significant differences were found in terms of “triple hit” or TMPRSS2:ERG expression. IHC analysis was feasible only in 17 patients due to insufficient material.

**Conclusions:**

CCP test may be a useful tool to estimate the risk of progression in PCa patients and guide the decision between AS and active treatment. Triple hit phenotype or TMPRSS:ERG fusion status was not associated with progression.

## 1 Introduction

Prostate cancer (PCa) is the most common solid neoplasia in the male and the second cause of cancer death in Western countries [1]. Early PCa diagnosis through PSA‐based screening has shown a significant reduction in PCa‐specific mortality [2] but may also lead to the overtreatment of indolent tumors that do not affect life expectancy or the quality of life. Low‐ and favorable intermediate‐risk PCa patients are candidates for active surveillance (AS) [3], and no survival difference between radical prostatectomy (RP) and simple observation has been demonstrated in these categories of patients [4, 5]. However, not all low‐risk tumors behave the same way: upgrading and upstaging at RP are still prevalent, indicating a potential biological aggressiveness in some cases [6, 7, 8]. A reliable tool for predicting disease progression remains an unmet clinical need, since the prognostic elements traditionally used until now have not proved sufficiently accurate to evaluate the behavior of the disease.

Some experimental biomarkers involving molecular gene analyses have been studied and developed in this context, but their use is not part of standard practice yet [9]. Among currently available tools, the cell‐cycle progression (CCP) test (Prolaris, Myriad Genetics, Salt Lake City, UT, USA) has been shown to be a predictor of cancer‐specific mortality and risk of metastases for PCa [10, 11, 12]. Prolaris was shown to improve the risk stratification of localized PCa patients [13, 14, 15] and to predict adverse pathology among men with clinically low‐risk PCa undergoing RP [16]. In parallel, other methods have been investigated, based on the immunohistochemical (IHC) analysis of PCa. In particular, Hernández‐Llodrà et al. found that a combination of ERG overexpression with PTEN and SLC45A3 (prostein) expression loss (a phenotype called “triple hit”) is associated with tumor progression and may represent another option to improve the risk category assignment [17]. Moreover, the expression levels of TMPRSS2‐ERG gene fusions have been related to a more aggressive PCa phenotype and could be molecular markers of progression [18, 19].

The aim of the present study was to assess and compare the potential of Prolaris, “triple hit” phenotype and TMPRSS2‐ERG expression for the early identification of progression in a cohort of patients with low‐ and favorable intermediate‐risk PCa under AS, to differentiate patients who will safely continue AS from those needing an active treatment.

## 2 Patients and Methods

### 2.1 Study Design and Data Collection

We performed a case‐control study, reviewing all consecutive patients who entered the AS programme at our Institution according to START criteria from January 2016 to August 2021 [20]. Men under AS that subsequently underwent RP for disease progression were considered as “cases”, while men still on AS at the end of the follow‐up period were labeled as “controls”. Medical records were searched, and patient characteristics (including a detailed medical history, PSA, digital rectal exam (DRE), MRI findings, and histopathological results) were recorded at diagnosis (baseline), during follow‐up, and at radical treatment, when applicable.

The study was conducted according to the Helsinki Declaration and all patients signed an informed consent for data collection and pathological and molecular analyses. No formal ethical committee approval was needed according to the Agenzia Italiana del Farmaco—AIFA guidelines for retrospective, observational studies. Our study design conformed to the Guidelines for the REporting of tumor MARKer Studies (REMARK) criteria for biomarker validation studies [21].

### 2.2 Patient Population and Inclusion Criteria

We included patients that entered AS following the protocol criteria of START, an observational no‐profit programme sponsored by Piedmont and Valle d'Aosta Oncology Network, enrolling low‐ and favorable intermediate‐risk patients [20]. START inclusion criteria were: (a) ISUP 1 PCa diagnosis at prostate biopsy in ≤ 2 cores (ISUP 2 accepted if aged ≥ 70 years; in case of MRI‐targeted biopsies, multiple cores aiming the same target counted as 1); (b) PSA ≤ 10 ng/mL; (c) fit for active treatment with a life expectancy of more than 10 years; (d) low risk of frailty/vulnerability; (e) signature of the informed consent. AS consisted of PSA measurement every 3 months, clinical visits every 6 months, and a rebiopsy after at least 1 year from diagnosis. Afterwards, rebiopsy was indicated at 3 and 5 years. A prostatic multiparametric MRI was recommended at diagnosis and during AS follow‐up. PSA increase or abnormal DRE were triggers for a repeat MRI and/or rebiopsy, at the discretion of the physician.

We enrolled as *controls* patients still under AS with a minimum 2‐year follow‐up, without signs of clinical progression and a rebiopsy after at least 1 year excluding PCa reclassification requiring active treatment. We enrolled as *cases* patients with documented disease progression during follow‐up, who subsequently underwent RP. Progression was defined as presence of ISUP ≥ 2 PCa if < 70 years; presence of ISUP ≥ 3 PCa for patients ≥ 70 years; ≥ 3 positive cores on biopsy. All RPs were performed with the Da Vinci robot. The presence of sufficient diagnostic biopsy histologic material was mandatory to perform molecular analyses.

### 2.3 Molecular (Prolaris) and IHC (Triple Hit) Analyses

The formalin‐fixed paraffin‐embedded (FFPE) diagnostic biopsies of all patients were retrieved from the Pathology Unit (Molinette Hospital) in Turin.

#### 2.3.1 CCP (Prolaris) Testing

Prolaris (Myriad Genetics, Salt Lake City, UT, USA) test kit utilizes quantitative RT‐PCR analysis to measure the RNA expression levels of 10 CCP genes and six housekeeper genes to generate a CCP Score from FFPE biopsy. The tumoral area of interest was first selected by a pathologist on Hematoxilin and Eosin slide, respecting a linear tumor length of 0.5 mm with ≥ 60% of tumoral tissue. Two to six sections were then cut with a thickness of 10 µm and retrieved in a 1.5 mL sterile tube. Total RNA was extracted post‐deparaffinization using a miRNeasy FFPE kit (QIAGEN, Hilden, Germany) according to the manufacturer's instructions. A quality control test was performed through PCR to determine whether RNA quality was good for the CCP Test. Subsequently, reverse transcription and pre‐amplification steps were performed on RNA, to prepare for the final quantitative PCR (qPCR). This allowed to determine RNA expression levels for a set of 16 predetermined genes: 10 CCP PCa‐related genes (*ASPM, CDC2, CDCA8, CDKN3, DTL, FOXM1, KIAA0101, NUSAP1, PRC1, TK1*) and six housekeeper genes (*CLTC, PSMA1, RPL4, RPS29, SLC25A3, UBA52*). All gene results were calibrated by their average expression in a set of commercial prostate tumor samples [10]. The Prolaris molecular score was defined as the average expression level of the CCP‐genes, normalized to the housekeeper genes. Finally, the Prolaris molecular score was combined with specific clinical variables at biopsy (including age, PSA, clinical T stage, percentage of positive cores, Gleason score and National Comprehensive Cancer Network risk) in a clinically validated and weighted algorithm to produce the Combined Clinical Risk (CCR) score and a suggestion for the subsequent treatment choice (Figure S1).

#### 2.3.2 IHC “Triple Hit” Testing

Three‐micron thick paraffin sections were processed using an automated platform (Ventana BenchMark AutoStainer; Ventana Medical Systems, Tucson, AZ) with antibodies against ERG (rabbit monoclonal antibody, clone EPR3864, Abcam, Cambridge, UK), prostein (SLC45A; mouse monoclonal antibody, clone 10E3, Dako Agilent, Santa Clara, USA), and PTEN (mouse monoclonal antibody, clone 6H2.1, Dako Agilent, Santa Clara, USA). Appropriate positive controls were included for each slide. Staining evaluation was assessed as previously described [17]. In particular, ERG was evaluated as positive or negative if nuclear staining was detected. A semiquantitative scoring system was used to evaluate prostein and PTEN as follows: 0 = total loss of expression, 1 = partial loss of expression and 2 = intense, homogeneous expression. A “triple hit” phenotype was diagnosed when PTEN and prostein showed at least a partial loss, accompanied by ERG overexpression.

#### 2.3.3 TMPRSS2:ERG Gene Fusions Expression Evaluation

RNAs extracted from the clinical samples were used to subtype them according to the presence/absence of the main genetic recombinations involving the ERG gene in PCa: TMPRSS2:ERG. Highly specific TaqMan probes (ThermoFisher) for Q‐PCR were used to subdivide the patients into two categories: TMPRSS2:ERG gene fusion positive (T1:E4+) and negative (T1:E4−).

### 2.4 Endpoint Definitions

The primary endpoint was to evaluate the ability at diagnostic biopsy of the Prolaris test (CCP and CCR scores) to detect PCa progression requiring active treatment during AS, distinguishing “cases” and “controls”. Secondary endpoint was to assess the impact on the risk of progression of molecular subtypes containing TMPRSS2:ERG gene fusion, and “triple hit” phenotype.

### 2.5 Statistical Analysis

Sample size calculation was based on the estimation that in recent years, in our institution, around 30 patients/year were enrolled in AS according to the START criteria [20]. Assuming from previous data that 30% of subjects experience disease progression during follow‐up, and estimating a population of 45 patients over 18 months, a sample size of 40 patients (12 cases and 28 controls) produces a two‐sided 95% confidence interval with a width equal to 30, from 16.6 to 46.5.

Statistical analyses were performed using SPSS, version 29 (IBM Corp., Armonk, NY, USA). Differences between groups were evaluated with the Median test and the Mann‐Whitney U test for continuous variables, *χ* ^2^ and the Fisher exact test for categorical variables. Uni‐ and multivariable logistic regression models were used to identify predictors of PCa reclassification, and their results were reported in terms of odds ratio and 95% confidence interval (CI). The multivariate model was built using traditional clinical variables (PSA, DRE, PIRADS score, ISUP grade) together with the prognostic variable that was statistically significant at univariate analysis. Statistical significance was considered at two‐sided *p* < 0.05.

## 3 Results

### 3.1 Patients' Characteristics

**Figure 1:**
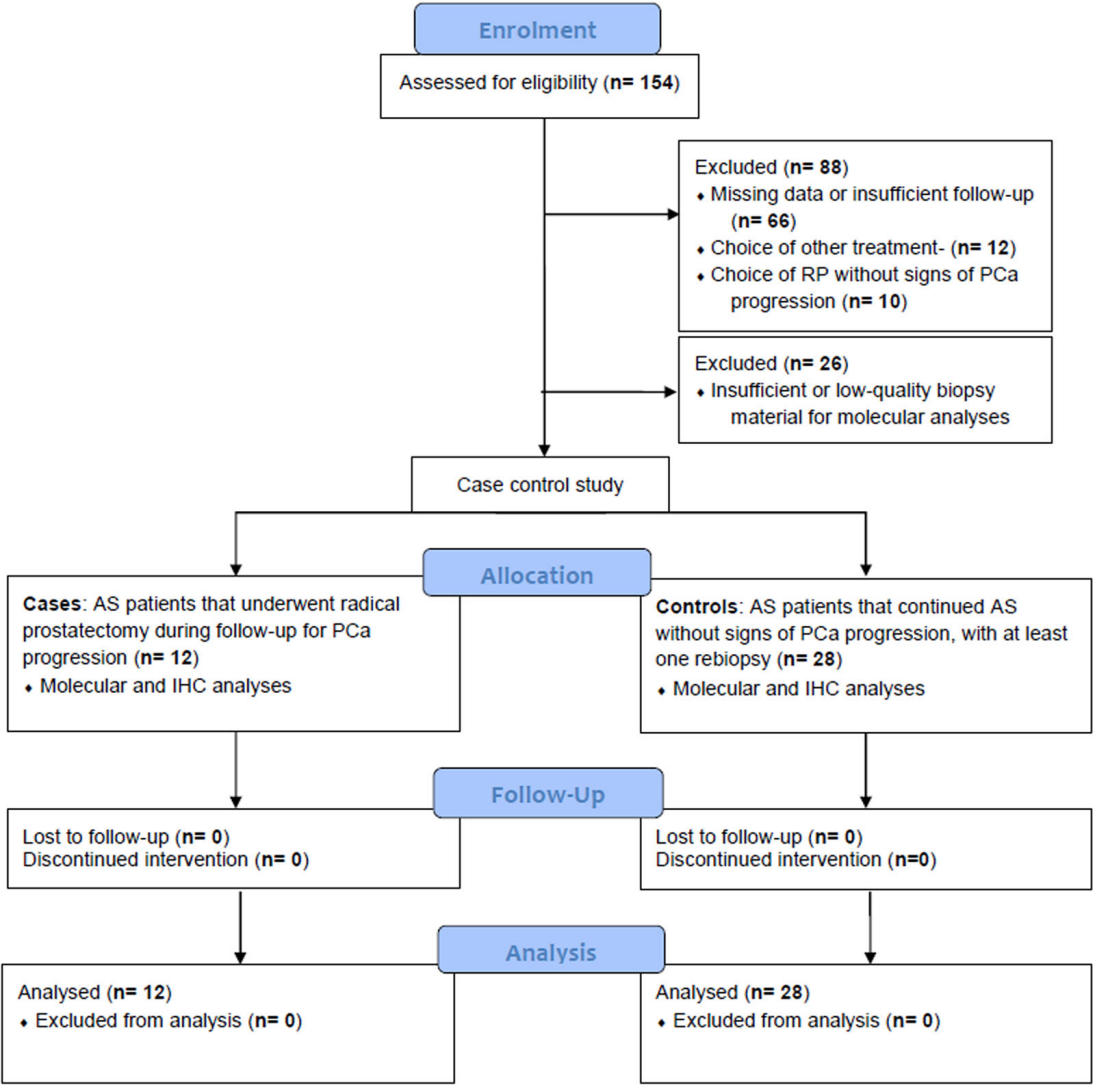
Study flowchart. [Color figure can be viewed at wileyonlinelibrary.com]

Among the 154 patients that initiated AS at our institution, we excluded 88 patients due to the following reasons: (1) incomplete clinical data; (2) unavailability of diagnostic biopsy; (3) AS patients with insufficient follow‐up or no rebiopsy; (4) AS patients that subsequently underwent radiotherapy; (5) AS patients that chose RP without signs of histopathological progression. After exclusion of 26 patients with insufficient or low‐quality biopsy material for molecular analyses, we finally included 40 patients (12 cases and 28 controls) in the study, as per sample size requirements (Figure 1).

Patients' characteristics are shown in Table 1. Clinical, radiological, and pathological characteristics were comparable between the two groups at the enrolment in AS, without significant differences in any of the considered variables, including the rate of patients with ISUP 2 PCa (35% in controls vs. 33% cases, *p* = 1.00). At diagnosis, the majority of patients received multiparametric prostatic MRI (87%) and MRI‐targeted biopsy plus a systematic mapping (70%). During follow‐up (39.5 months on average), the group of cases experienced higher rates of PSA increase > 50% (33% vs. 14%, *p* = 0.21) and disease progression visible at MRI (80% vs. 42%, *p* = 0.11), but none of these differences were statistically significant. Significantly higher rates of ISUP grade progression (83% vs. 3%, *p* < 0.001) and increase in positive cores (75% vs. 10%, *p* < 0.001) at rebiopsy were recorded in the group of cases. After RP, most cancers were scored as ISUP grade group 2 (*n* = 8) while the remaining were grade 3 (*n* = 4).

**Table 1:** Patients' characteristics.

Characteristics at enrolment in AS	Overall	Controls (continued AS)	Cases (switched to treatment)	*p* value
Number of patients	40	28	12	—
Age, year, median (IQR)	70 (65–74)	71 (67–74)	66 (60–74)	0.40
PSA, ng/mL, median (IQR)	5.9 (5.3–7.5)	5.8 (5.1–7.2)	6.9 (5.7–9.3)	0.73
Previous negative prostate biopsy, n (%)	10 (25.0%)	6 (21.4%)	4 (33.3%)	0.45
Prostate volume, cc, median (IQR)	54.0 (40.2–64.8)	57.9 (44.2–64.7)	51.0 (32.6–64.8)	0.53
PSA density, cc, median (IQR)	0.11 (0.07–0.15)	0.10 (0.07–14.8)	0.13 (0.10–0.17)	0.11
Clinical T stage, *n* (%)				1.00
cT1c	29 (72.5%)	20 (71.4%)	9 (75.0%)	
cT2a	11 (27.5%)	8 (28.6%)	3 (25.0%)	
MRI performed, *n* (%)	35 (87.5%)	25 (89.3%)	10 (83.3%)	0.62
Max PIRADS score, *n* (%)				0.30
2	1 (2.9%)	0 (0%)	1 (10.0%)	
3	4 (11.4%)	2 (8.0%)	2 (20.0%)	
4	19 (54.3%)	15 (60.0%)	4 (40.0%)	
5	2 (5.7%)	2 (8.0%)	0 (0.0%)	
Not specified	9 (25.7%)	6 (24.0%)	3 (30.0%)	
Target diameter^a^, mm, median (IQR)	10.0 (6.0–11.0)	10 (6.0–11.5)	10.0 (7.0–11.0)	0.66
Type of biopsy, *n* (%)				0.23
Targeted + systematic	28 (70.0%)	21 (75.0%)	7 (58.3%)	
Systematic only	11 (27.5%)	7 (25.0%)	4 (33.3%)	
Targeted only	1 (2.5%)	0 (0%)	1 (8.3%)	
ISUP grade at biopsy, *n* (%)				1.00
1	26 (65.0%)	18 (64.3%)	8 (66.7%)	
2	14 (35.0%)	10 (35.7%)	4 (33.3%)	
Positive biopsy cores^b^, *n*, median (IQR)	1.0 (1.0–2.0)	1.0 (1.0–2.0)	1.0 (1.0–2.0)	0.83
Targeted biopsy cores, *n*, median (IQR)	3.0 (0.25–4.0)	3.0 (0.25–4.0)	3.0 (0.50–3.75)	0.76
Systematic biopsy cores, *n*, median (IQR)	12.0 (11.0–12.0)	12.0 (11.0–12.0)	12.0 (11.2–12.0)	0.65
Total biopsy cores, *n*, median (IQR)	15.0 (12.0–15.0)	15.0 (12.0–15.0)	13.5 (12.0–15.7)	0.93

**Follow‐up**	**Overall**	**Controls**	**Cases**	***p***
Follow‐up, months, mean (SD)	39.5 (22.3)	36.6 (21.6)	46.4 (23.3)	0.20
PSA increase > 50%, *n* (%)	8 (20.0%)	4 (14.3%)	4 (33.3%)	0.21
MRI progression, *n* (%)	16 (55.2%)	8 (42.1%)	8 (80.0%)	0.11
ISUP grade progression at last rebiopsy, *n* (%)	11 (27.5%)	1 (3.6%)	10 (83.3%)	< 0.001
Increase of positive cores at last rebiopsy, *n* (%)	12 (30.0%)	3 (10.7%)	9 (75.0%)	< 0.001
ISUP grade at radical prostatectomy, *n* (%)	—	—		—
2			8 (66%)	
3			4 (33%)	
TNM at radical prostatectomy, *n* (%)	—	—		—
pT2b/c Nx/0			11 (91.7%)	
pT3a N1			1 (8.3%)	
pR0			12 (100%)	

Abbreviations: AS, active surveillance; ISUP, International Society of Urological Pathology; MRI, magnetic resonance imaging; PIRADS, prostate imaging reporting and data system.

^a^ In case of two targets in the same patient, a larger MRI target diameter is considered.

^b^ Multiple positive target cores counted as one.

### 3.2 Molecular and IHC Results

Table 2 shows the results of molecular and IHC analyses in cases and controls. The indication of CCP test suggested to pursue AS in 75% of controls, while overestimating the switch to active treatment in 25% of patients who continued AS without signs of progression at a mean follow‐up of 36.6 months. The indication of CCP test suggested an active treatment in 75% of cases, while suggested to pursue AS in 25% of patients who underwent RP during their follow‐up due to disease progression. CCP test suggested active treatment in the only patient staged as pT3a after RP. All the other patients undergoing surgery had localized disease.

**Table 2:** Results of molecular and IHC analyses in cases and controls.

	Controls (*n* = 28) (continued AS)	Cases (*n* = 12) (switched to treatment)	*p*
Prolaris test indication, *n* (%)			
AS	21/28 (75.0)	3/12 (25.0)	0.005
Treatment	7/28 (25.0)	9/12 (75.0	
Prolaris molecular score, mean (SD)	3.81 (0.67)	4.80 (0.85)	< 0.001
Prolaris combined clinical risk score, mean (SD)	0.59 (0.46)	1.21 (0.52)	0.001
Cancer specific mortality risk predicted by Prolaris test, mean (SD)	1.88 (1.77)	4.75 (1.94)	< 0.001
Complete or partial PTEN loss^a^, *n* (%)	9/12 (75)	5/5 (100)	0.51
Complete or partial prostein loss^a^^,^ b, *n* (%)	2/11 (18.2)	3/5 (60)	0.24
ERG overexpression^a^, *n* (%)	2/12 (16.7)	0/5 (0)	1.00
Triple hit^a^, *n* (%)	2/12 (16.7)	0/5 (0)	1.00
TMPRSS2:ERG expression			
Positive (T1/E4+)	10 (35.7)	6 (50.0)	0.39
Negative (T1/E4−)	18 (64.3)	6 (50.0)	

Abbreviation: AS, active surveillance.

^a^ 23 missing due to insufficient FFPE material for IHC.

^b^ 1 failed analysis.

**Figure 2:**
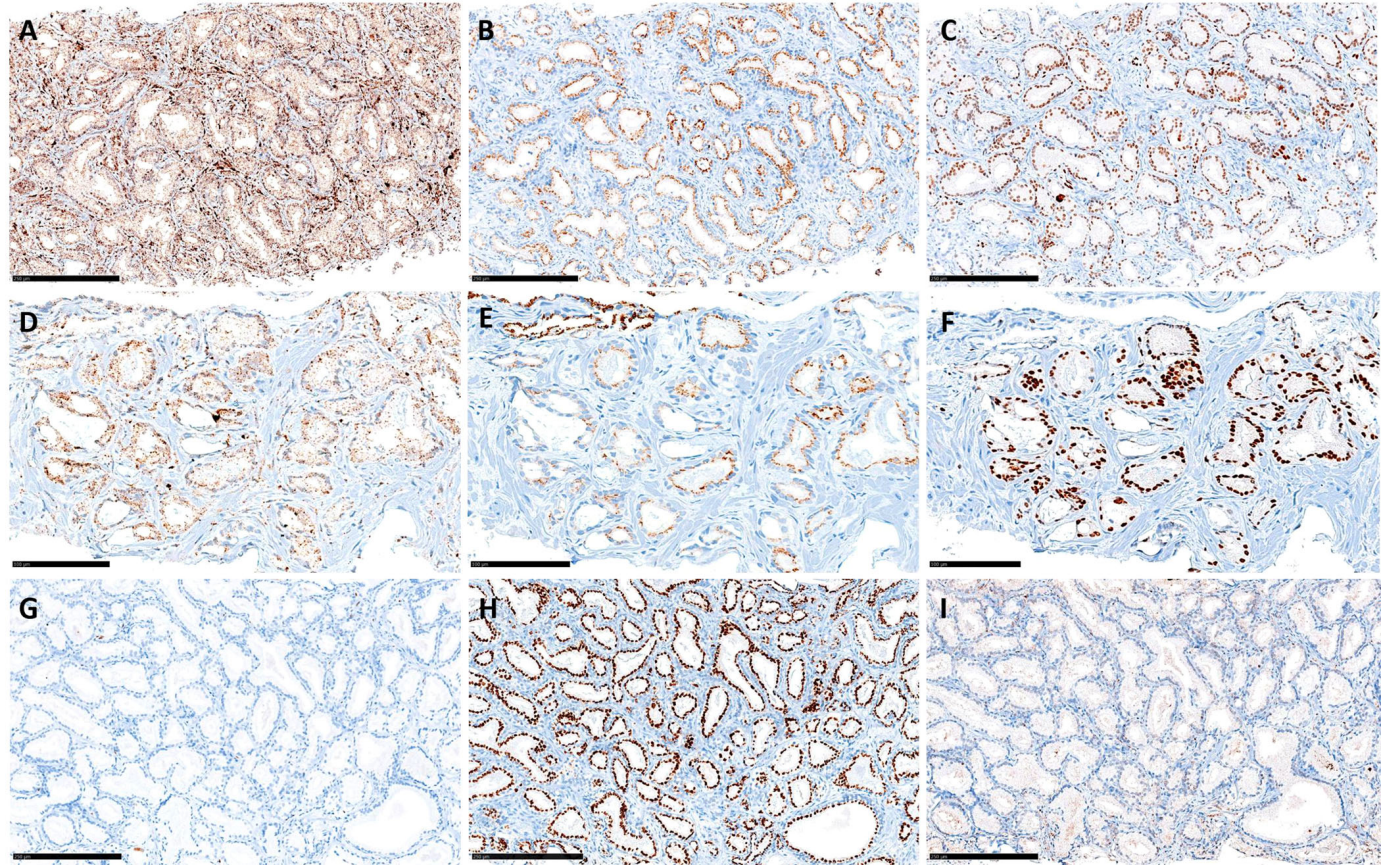
Immunohistochemistry of ERG, Prostein (SLC45A3), and PTEN in three cases of prostate cancer. Case 1, PTEN intense and homogeneous expression (A; 15×), Prostein partial loss of expression (B; 15×), and ERG positive immunostaining (C; 15×). Case 2, PTEN partial loss of expression (D; 20×), Prostein partial loss of expression (E; 20×), and ERG positive immunostaining (F; 20×). Case 3, PTEN total loss of expression (G; 15×), Prostein intense and homogeneous expression (H; 15×), and ERG negative immunostaining (I; 15×). [Color figure can be viewed at wileyonlinelibrary.com]

IHC was feasible only in 17 patients (12 cases and 5 controls) due to insufficient remaining FFPE material for the analysis. With this limitation, the “triple hit” was not able to differentiate between cases and controls, nor were PTEN loss, prostein loss, or ERG overexpression taken singularly. Figure 2 shows different results of ERG, PTEN, and prostein expression in three of the analyzed cases. Most analyzed patients had complete or partial PTEN loss (5/5 cases and 9/12 controls), while ERG overexpression was found only in two patients, both belonging to the controls.

By Q‐PCR we identified 16 TMPRSS2:ERG‐positive patients. The highest and lowest ERG and PTEN expression levels were found in TMPRSS2:ERG‐positive patients undergoing active treatment (Figure S2). No significant difference was found between cases and controls in terms of TMPRSS2:ERG expression. When analyzing the performance of CCP test according to TMPRSS2:ERG expression, we found that CCP test overestimated the switch to active treatment in 33.3% of the TMPRSS2:ERG‐negative and 10% of the TMPRSS2:ERG‐positive patients, while it correctly estimated the need for active treatment in 83.3% and 66.7% in TMPRSS2:ERG‐negative and TMPRSS2:ERG‐positive patients, respectively (Table S1).

### 3.3 Logistic Regression Analyses

The logistic regression (Table 3) confirmed the role of the Prolaris test to detect cancer progression. Prolaris molecular score (HR 7.1, 95% CI 1.7–28.7, *p* = 0.006), Prolaris CCR score (HR 11.4, 95% CI 2.1–60.8, *p* = 0.004), and the Prolaris indication (HR 9.0, 95% CI 1.8–35.3, *p* = 0.006, intended as a categorical variable) were associated with progression at univariable analysis. Prolaris molecular score was also associated with progression at multivariable analysis (HR 8.5, 95% CI 1.2–58.8, *p* = 0.02). During follow‐up, progression of ISUP grade at rebiopsy was the only variable associated with disease progression (HR 135, 95% CI 10.9–1657.3, *p* < 0.001), as opposed to PSA increase > 50% or MRI progression. Neither ERG nor PTEN expression levels were associated to disease progression in all 40 patients (HR 0.07, 95% CI 0.002–2.3, *p* = 0.13 and HR 0.08, 95% CI 4.95E‐09–1.28E06, *p* = 0.76), respectively), in the TMPRSS2:ERG‐negative subgroup (HR 0.04, 95% CI 7.73E‐09–3073.03, *p* = 0.43 and HR 0.0004, 95% CI 7.72E‐16–1.99E‐08, *p* = 0.57) or in the TMPRSS2:ERG‐positive subgroup (HR 0.013, 95% CI 6.0–2.71, *p* = 0.11 and HR 1.75, 95% CI 2.90E‐09–1.0E‐09, *p* = 0.95).

**Table 3:** Univariate and multivariate analyses on PCa progression of AS patients.

	Univariate		Multivariate model	
Variable	OR (95% CI)	*p*	OR (95% CI)	*p*
Variables at diagnosis				
PSA	1.3 (0.8–1.9)	0.17	0.9 (0.5–1.6)	0.81
Positive digital rectal examination	0.8 (0.1–3.8)	0.81	2.2 (0.0–58.8)	0.63
PIRADS score 4 or 5	0.15 (0.0–1.2)	0.08	0.1 (0.0–2.1)	0.16
ISUP grade at biopsy		0.88		0.14
1	Ref		Ref	
2	0.9 (0.2–3.7)		0.1 (0.0–2.1)	
Prolaris molecular score	7.1 (1.7–28.7)	0.006	8.5 (1.2–58.8)	0.02
Combined clinical risk score	11.4 (2.1–60.8)	0.004	—	—
Prolaris test indication	9.0 (1.8–35.3)	0.005	—	—
ERG overexpression	0.07 (0.002–2.30)	0.13	—	—
PTEN loss	0.08 (4.95E‐09–1.28E06)	0.76	—	—
Variables during follow‐up				
PSA increase > 50%	3.0 (0.6–14.8)	0.17	—	—
MRI progression	5.5 (0.9–33.1)	0.06	—	—

## 4 Discussion

In the everyday clinical practice, the decision between AS, focal therapy, or radical treatment still relies on traditional clinical parameters, including PSA level, DRE and MRI findings, and ISUP score [3]. However, these parameters are often unreliable to predict disease progression in case of low‐ and favorable‐intermediate risk PCa, as demonstrated by the non‐negligible proportion of patients initially put under AS who progress in the first years, switching to active treatment [22, 23]. The PRIAS study, which enrolled 5302 men with ISUP 1 PCa under AS, registered a 34% discontinuation at 5 years because of protocol‐based reclassification, namely disease progression [22]. The reclassification from ISUP 1 to ISUP ≥ 2 was 21% at 5 years in a large prospective cohort of very‐low or low‐risk patients monitored on AS at Johns Hopkins [23]. Nowadays patients' selection is more accurate thanks to the MRI guidance for the diagnostic biopsy, which limits the risk of PCa understaging or undergrading. Nevertheless, it is hard to understand why some patients progress while others safely remain under AS. In this setting, some commercially available molecular biomarkers, such as the Decipher genomic classifier, the Prolaris CCP test, and the Oncotype Dx test, might help stratify patients according to the risk of an adverse oncological event in the future and thereby help in treatment selection. However, their actual role in the clinical practice and their impact on clinical, oncological, and functional outcomes are unclear, and their routine use is not recommended by urological guidelines [9].

In the current study we focused on three different tools to test their ability in assess the risk of progression in a homogeneous series of PCa patients under AS, of whom we already knew the clinical course: 28 *controls* continued monitoring without any trace of progression, confirmed by at least one rebiopsy during follow‐up, and 12 *cases* were switched to RP for documented histological disease progression. To our knowledge, this is the first study in the literature with such a design. As expected, none of the traditional clinical variables was able to correctly evaluate the risk of progression, not even the PSA trend or the MRI findings during follow‐up.

Prolaris test was strongly associated with progression at logistic regression, both as a raw molecular score and when integrated in the CCR score. The indication of Prolaris test to pursue AS was correct in 75% of our *controls*, as well as in 75% of our *cases* for whom an indication for active treatment was given. There remains a gray zone, represented by a minority of patients where Prolaris test over‐ or underestimated the outcome after a mean follow‐up of 39 months. All our patients who needed RP had localized disease, except for one who after surgery was diagnosed with extracapsular disease and nodal involvement, not detected by MRI. In this case, the Prolaris test performed on diagnostic biopsy would have correctly suggested an immediate active treatment. Four out of our 12 *cases* progressed to ISUP 3; of them, three were selected by Prolaris test for an active treatment. In 2021, Cooperberg et al. conducted a case‐control study on 641 low‐risk, ISUP 1 patients undergoing RP. Of them, 236 had minor upstaging/upgrading (ISUP 2 or pT3a) and 67 had major upstaging/upgrading (ISUP ≥ 3 or ≥ pT3b and CCP score obtained from ISUP 1 tissue was directly associated with minor or major events on univariate and multivariate analyses, suggesting a predictive role of Prolaris test in this setting of patients [16]. Of note, a major limitation of this study was the use of virtual biopsies from RP tissue as surrogates for prostate biopsies, limiting their applicability in the clinical practice. Our study overcomes this limitation, having analyzed PCa tissue obtained from diagnostic biopsies. On the other hand, it raises the issue of the feasibility of these tests in low‐risk patients, where the amount of PCa found at biopsy is usually scarce, by definition, based on the study's inclusion criteria. When selecting suitable patients for our study, 26 were excluded due to insufficient or low‐quality material for molecular analysis. Therefore, not all patients could benefit from the use of a molecular test to guide the decision between AS or active treatment for feasibility issues.

As for IHC analyses we could not detect any difference between *cases* and *controls* by testing PTEN, prostein, or ERG. The feasibility issue here was even more relevant, as only 17 out of 40 patients could be tested due to insufficient remaining FFPE material for the analysis. In 2017, Hernández‐Llodrà et al. found that in ISUP 1 PCa, single ERG positive immunostaining was associated with progression, and the combination with PTEN and prostein loss (the so‐called “triple hit”) was strongly linked to both adverse pathological features and PSA progression‐free survival [17]. However, we could not confirm the clinical use of these biomarkers in low‐risk PCa patients to guide the treatment decision.

Finally, great interest has arisen around the potential predictive role of TMPRSS2:ERG gene fusions that have been related to a more aggressive PCa phenotype [18, 19]. ERG overexpression is associated with advanced PCa stage, elevated ISUP score, metastasis, and reduced survival [24]. In around half of PCa patients, illegitimate recombination of ERG with other genes is present, with TMPRSS2:ERG representing the dominant fusion [25]. Fusion status is a key genomic event, but its prognostic value has not been proven yet. In 2014, Berg et al. investigated the ERG IHC expression on biopsies of 265 patients in AS, finding that ERG‐positive patients had a significantly higher risk of disease progression compared with fusion‐negative patients [26]. The 2‐year cumulative incidence of overall progression was 22%, comparable to other series in the literature. The overexpression of ERG is highly attributed to the TMPRSS2:ERG gene fusion, but its prognostic utility remains debated [19]. Our study did not detect any difference between patients with or without TMPRSS2:ERG fusion, maybe due to the small sample size. Interestingly, the prognostic role of ERG or PTEN expression did not change according to the presence of TMPRSS2:ERG recombination. The incidence of TMPRSS2:ERG gene fusion found in our molecular analyses is in line with the literature, whereas the rate of our ERG IHC expression was unexpectedly low, possibly due to false negatives, considering also the low amount of FFPE material available for the analysis.

Our study is not devoid of limitations, mainly residing in its retrospective nature and the small sample size. In particular, the IHC was feasible only in a smaller subgroup of patients than that originally planned. Despite not being a prospective randomized trial, it was specifically designed for the comparison of the two groups in the study, expecting a progression rate according to the data gathered by our regional study on low‐risk PCa patients. The difficulty in performing molecular and IHC analyses reflects a real‐world issue in this setting of patients.

## 5 Conclusion

CCP Prolaris test was associated with cancer progression in low‐ and favorable intermediate risk PCa patients and could be a useful tool to guide the decision between AS and active treatment. Triple hit phenotype or TMPRSS:ERG fusion status was not associated with progression. Molecular and IHC analyses are not always feasible in this setting of patients due to the insufficient amount or quality of tumoral tissue.

### 5.1 Take Home Message

Prolaris cell‐cycle progression test score was associated with prostate cancer progression and might help to guide the decision between active surveillance and active treatment.

## Author Contributions

Study design: Marco Oderda, Paola Francia di Celle, Ian Marc Bonapace, Mauro Papotti, Paolo Gontero. Data acquisition: Giorgio Calleris, Daniele D'Agate, Gabriele Montefusco. Pathological and molecular analysis: Giulia Orlando, Giulia Capella, Luisa Delsedime, Eleonora Duregon, Paola Francia di Celle, Donatella Pacchioni, Ian Marc Bonapace, Zaibunnisa Zaibunnisa. Analyzing data: Marco Oderda, Claudia Filippini, Ian Marc Bonapace. Drafting the manuscript: Marco Oderda, Giorgio Calleris, Giulia Orlando, Ian Marc Bonapace. All authors were involved in the manuscript revision for important intellectual content.

## Ethics Statement

The study was conducted according to the Helsinki Declaration, and all patients signed an informed consent for clinical and pathological data collection, and for the performance of genomic analyses. No formal ethical committee approval was needed according to the Agenzia Italiana del Farmaco—AIFA guidelines for observational studies.

## Conflicts of Interest

The authors declare no conflicts of interest.

## Supporting information


**Supplementary table 1.** Indications of Prolaris test according to the presence of TMPRSS:ERG recombination.

**Supporting figure 1**. Prolaris report suggesting the treatment choice.

**Supporting figure 2**. Violin plot of the expression levels (*z*‐score) of ERG and PTEN in all patients (All) and in TMPRSS2:ERG positive (T1:E4+) and negative (T1:E4−) patients. Abbreviations: AS, active surveillance; AT, active treatment. *p* Value: **, ≤ 0.01.

## Data Availability

The data that support the findings of this study will not be published in a repository and are available from the corresponding author, M.O., upon reasonable and motivated request.
