# Supplementary material for: Role of Cell‐Cycle Proliferation Test, Triple Hit Phenotype, and TMPRSS2‐ERG Expression to Evaluate the Risk of Progression in Prostate Cancer Patients Under Active Surveillance
Source: Prostate. 2025 May 29;85(12):1104–13. doi: 10.1002/pros.24921 (PMC12278703; doi:10.1002/pros.24921)
Supplement: Supplementary file 1 — Supplementary table 1. Indications of Prolaris test according to the presence of TMPRSS:ERG recombination. [file PROS-85-1104-s002.docx]

**Supplementary table 1. Indications of Prolaris test according to the presence of TMPRSS:ERG recombination**

| **Presence of TMPRSS:ERG recombination (T1:E4+)** | **Controls (n=10)**  **(continued AS)** | **Cases (n=6)**  **(switched to treatment)** | **p** |
| --- | --- | --- | --- |
| Prolaris test indication   - AS - Treatment | 9 (90.00%)  1 (10.00%) | 2 (33.33%)  4 (66.67%) | 0.036 |
| **Absence of TMPRSS:ERG recombination (T1:E4-)** | **Controls (n=18)**  **(continued AS)** | **Cases (n=6)**  **(switched to treatment)** | **p** |
| Prolaris test indication   - AS - Treatment | 12 (66.67%)  6 (33.33%) | 1 (16.67%)  5 (83.33%) | 0.061 |
