# Supplementary figures and images for: Role of Cell‐Cycle Proliferation Test, Triple Hit Phenotype, and TMPRSS2‐ERG Expression to Evaluate the Risk of Progression in Prostate Cancer Patients Under Active Surveillance
Source: Prostate. 2025 May 29;85(12):1104–13. doi: 10.1002/pros.24921 (PMC12278703; doi:10.1002/pros.24921)

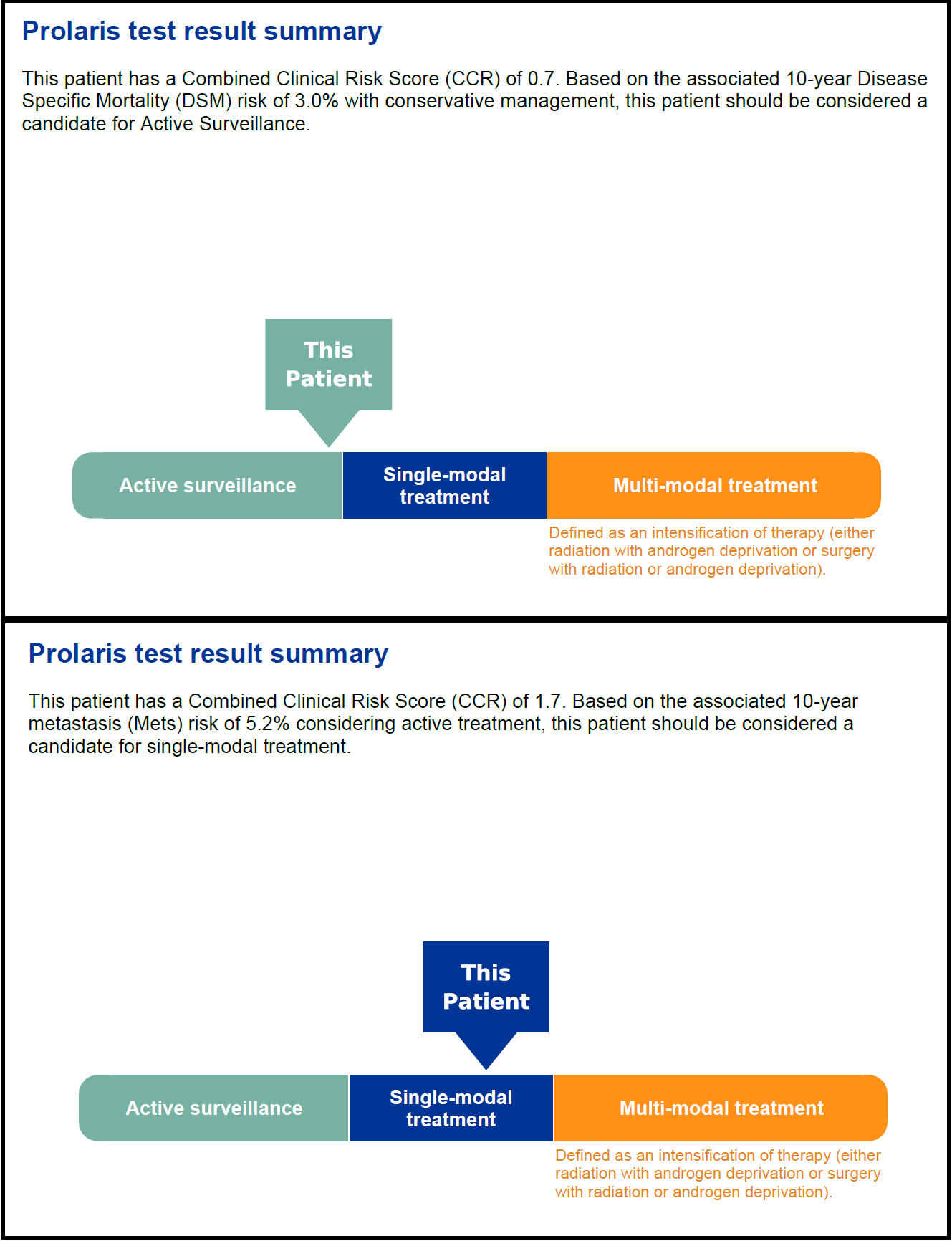

Supplement: Supplementary file 2 — Supporting figure 1. Prolaris report suggesting the treatment choice. [file PROS-85-1104-s003.tif]

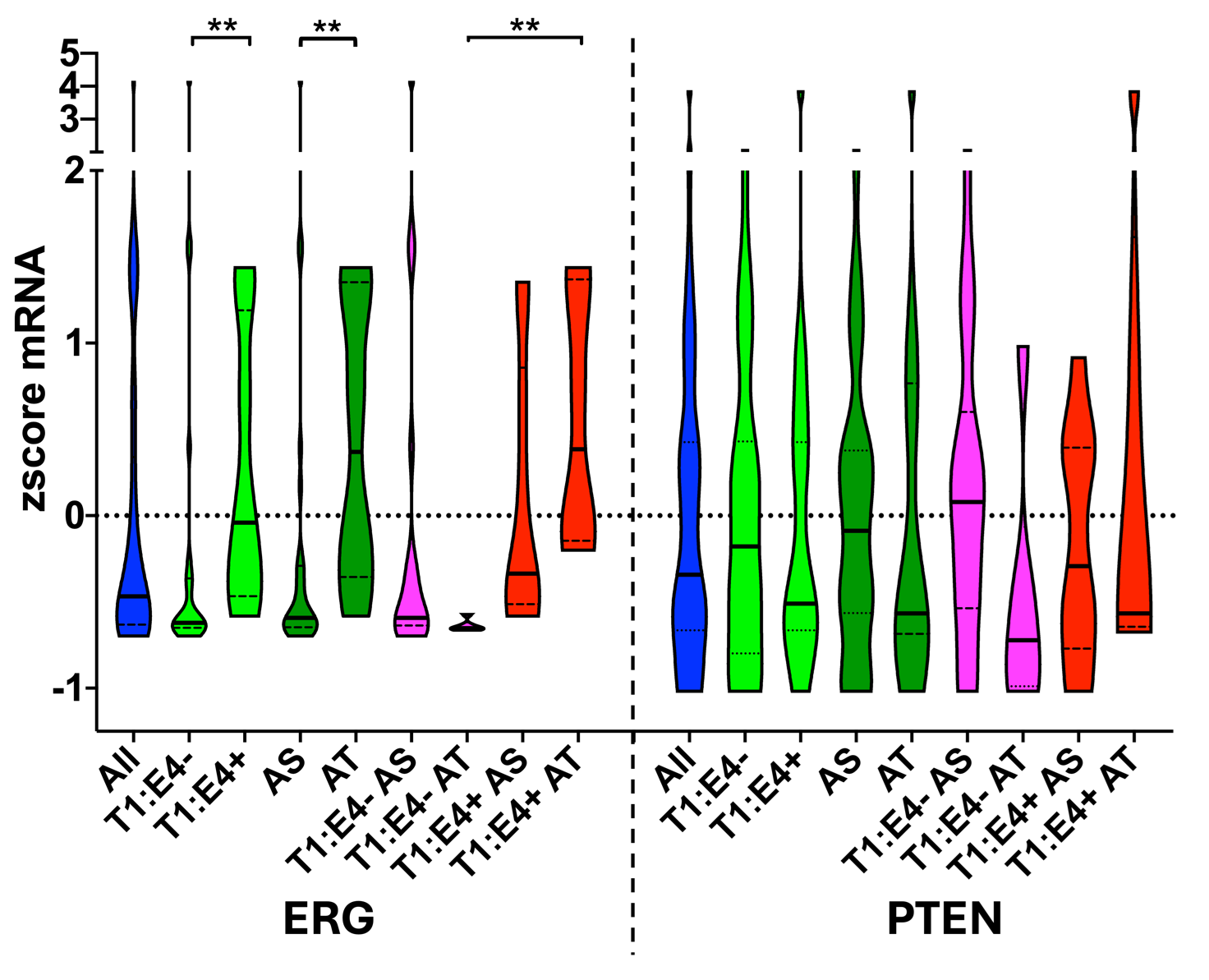

Supplement: Supplementary file 3 — Supporting figure 2. Violin plot of the expression levels (z‐score) of ERG and PTEN in all patients (All) and in TMPRSS2:ERG positive (T1:E4+) and negative (T1:E4−) patients. Abbreviations: AS, active surveillance; AT, active treatment. p Value: **, ≤ 0.01. [file PROS-85-1104-s001.tif]
